# Variable Impedance Control Based on Target Position and Tracking Error for Rehabilitation Robots During a Reaching Task

**DOI:** 10.3389/fnbot.2022.850692

**Published:** 2022-03-03

**Authors:** Rongrong Tang, Qianqian Yang, Rong Song

**Affiliations:** ^1^The Key Laboratory of Sensing Technology and Biomedical Instrument of Guangdong Province, School of Biomedical Engineering, Sun Yat-sen University, Guangzhou, China; ^2^The Shenzhen Research Institute, Sun Yat-sen University, Guangzhou, China; ^3^The School of Mechatronic Engineering, Guangdong Polytechnic Normal University, Guangzhou, China

**Keywords:** rehabilitation robot, reaching task, variable impedance, target position, tracking error, smooth trajectory

## Abstract

To obtain an anthropomorphic performance in physical human-robot interaction during a reaching task, a variable impedance control (vIC) algorithm with human-like characteristics is proposed in this article. The damping value of the proposed method is varied with the target position as well as through the tracking error. The proposed control algorithm is compared with the impedance control algorithm with constant parameters (IC) and another vIC algorithm, which is only changed with the tracking error (vIC-e). The different control algorithms are validated through the simulation study, and are experimentally implemented on a cable-driven rehabilitation robot. The results show that the proposed vIC can improve the tracking accuracy and trajectory smoothness, and reduce the interaction force at the same time.

## Introduction

Stroke has become a leading cause of death and disability throughout the world (Kim et al., 2020). Research has shown that consistent and repeated rehabilitation training has a positive effect on the motor function recovery of stroke patients (Kwakkel, 2006). Traditional rehabilitation therapies carried out by medical therapists are usually labor intensive. Due to this, rehabilitation robots have come to play an increasingly pivotal role in relation to motor function recovery and cortical reorganization (Jiang et al., 2022).

Previous studies have shown that the robot with a compliant control algorithm performs better rehabilitation training for its natural human-robot interaction (HRI) (Niu et al., 2017; Yang et al., 2020). Since pure force or position control, like that exhibited by the Proportion-Integration-Differentiation (PID) control algorithm (Rosati et al., 2007), has proved inadequate for compliant interaction, Hogan (1985) have proposed an impedance control method which took into account the mechanical interaction between systems and the environment (Veneman et al., 2007). Otten et al. (2015) have reported that an upper-limb exoskeleton moved more smoothly with the impedance control. In uncertain environments or challenging design tasks, the impedance control algorithm based on constant parameters may lead to increased difficulty in terms of human-robot cooperation (Yang C. G. et al., 2018; Li et al., 2019). In the recent years, variable impedance control has received increased attention owing to its effective interaction with different environments (Al-Shuka et al., 2018; Dong et al., 2019). The impedance parameters are usually adjusted by the kinematic or dynamic information measured in the interactive tasks (Lee et al., 2014). Ficuciello et al. (2015) modulated the damping value based on the velocity of the end effector, and found that the variable impedance control could reach a compromise between task accuracy and execution time. Stegall et al. (2017) adjusted the damping value according to the tracking error, and improved the tracking ability. Li et al. (2018) varied the damping value using the force exerted by the subject to provide a stable motion transition and compliant HRI. Variable impedance control has a good application in rehabilitation robots for its high flexibility and adaptivity.

It is known that human movements are characterized by high efficiency and adaptation, which might be result from the variable limb impedance parameters (Burdet et al., 2001). The mechanical impedance of the human arm composed of inertia, damping, and stiffness. They are affected by the effects of co-contraction of antagonistic muscles, involuntary reflexes, and the inherent viscoelasticity of limbs (Gomi and Osu, 1998); and they are also decided by the neuromuscular characteristics of the limbs and the motion commands from the central nervous system (Dolan and Friedman, 1993). Many researchers have measured the impedance of a human limb when completing a specific task. Erden and Billard (2015) measured the impedance by introducing external force disturbances and fitting the human hand response to a mass-damping-spring model. They found that the impedance parameters changed with the external force and the induced arm displacement. Identifying the distance between the arm displacement and the targets is essential for the reaching tasks (Hornsey and Hibbard, 2021). The impedance parameters of the human arm are also related to the tracking error rate in order to ensure accurate movements (Yang et al., 2011). Another characteristic of human movement is smooth motion trajectory (Hogan, 1984). The models of the motion trajectory characteristics include the minimum torque model (Uno et al., 1989), minimum squared derivative principles (Pham et al., 2007), etc. It has been hypothesized that maximizing the smoothness is obtained by minimizing the squared jerk. The minimum jerk (MJ) model has successfully predicted the characteristics of upper limb movements with bell-shaped velocity profiles (Bizzi et al., 1984; Flash and Hogan, 1985). Since then, the MJ model has been widely used to obtain naturalistic and smooth upper arm motion.

In previous studies, variable impedance control algorithms have rarely taken the real-time target position into account during reaching tasks. Though they can obtain a compromise between tracking accuracy and adaption, the compliance of HRI still needs to be improved. On the other hand, the control algorithms usually focus on the variable impedance or the smooth trajectory, separately (Sidobre and Desornneaux, 2019). In addition, the commonly used reference trajectories of rehabilitation robots, such as sinusoidal trajectories (Yang X. et al., 2018), is inconsistent with upper limb motion, and may cause an impact on the subject.

A variable impedance control is proposed in this article. The variable damping value is varied according to the tracking error and the target position at the same time. A reaching task with a MJ trajectory as the reference trajectory is used to validate the proposed method. Simulations and experiments have been conducted on an upper-limb cable-driven rehabilitation robot (CDRR). The proposed variable impedance control is compared with the impedance control with constant parameters and a variable impedance control that is usually used.

## Methods

**Figure 1:**
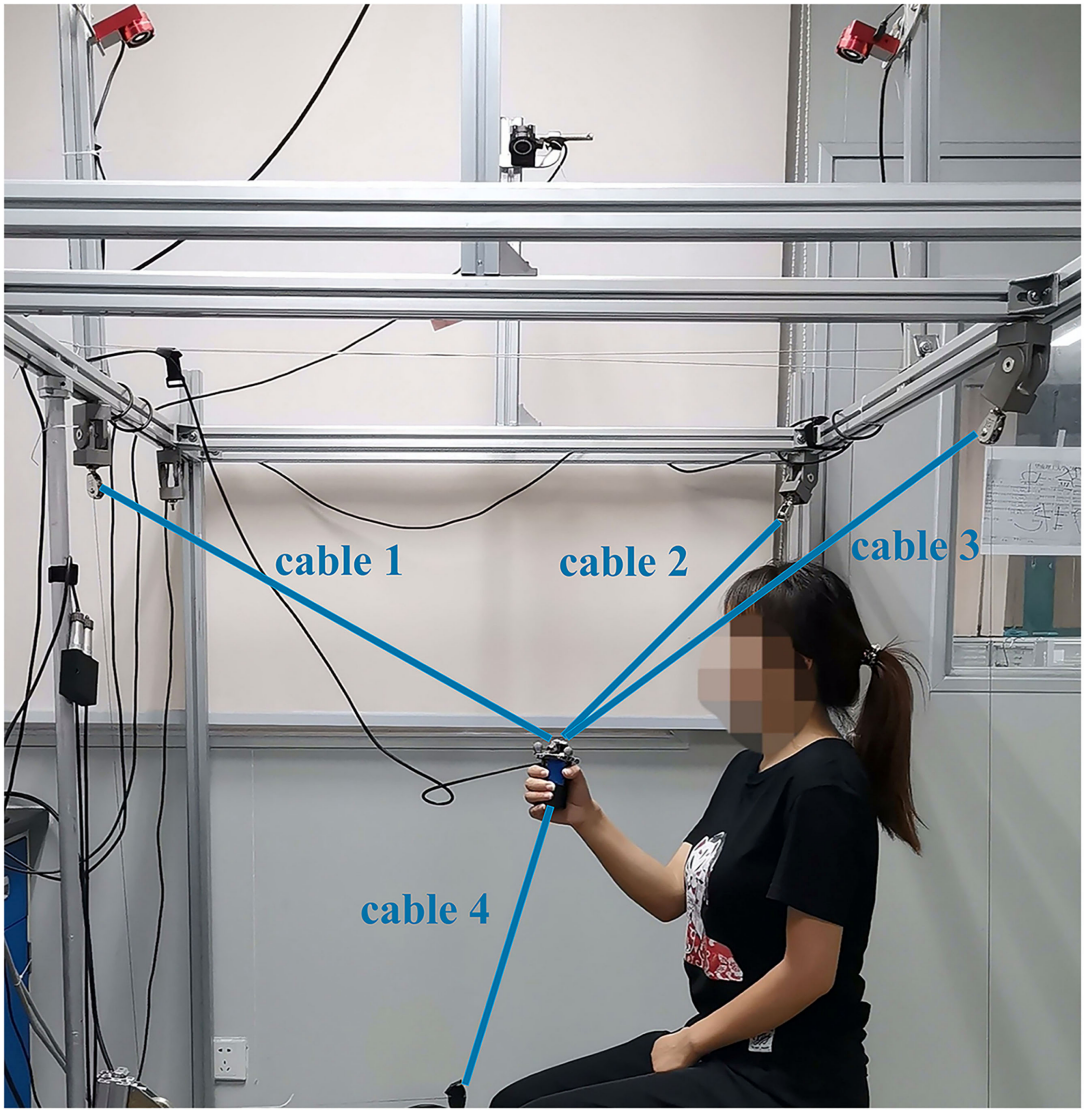
The schematic representation of the cable-driven rehabilitation robot (CDRR).

In this article, the HRI system included a subject and a cable-driven rehabilitation robot. The four-cable CDRR, as shown in Figure 1, was developed with the upper three cables for motion and the redundant downward fourth cable for stabilization (Li et al., 2021). The CDRR was designed for the rehabilitation of the shoulders and elbows, and it had three translational degrees of freedom. A controller (MicroLabBox, dSPACE, Germany) and four servo motors (DM1B-045G, Yokogawa, Japan) were used as the main mechanical part with the four cables. The tensile force of each cable could be measured by S-shaped tensile force sensors. In order to obtain the position of the end effector in real time, a motion capture system (OptiTrack, NaturalPoint, USA) with four infrared cameras around the subjects was utilized. To decrease the effect of the end-effector rotation during the movement, three markers placed on the end effector was used to calculate the position of the center point, which was regarded as the position of the end effector. The data of the controller and motion capture system were recorded at a frequency of 100 Hz. A safe button was used for emergency stop during the experiment.

### Control Law

**Figure 2:**
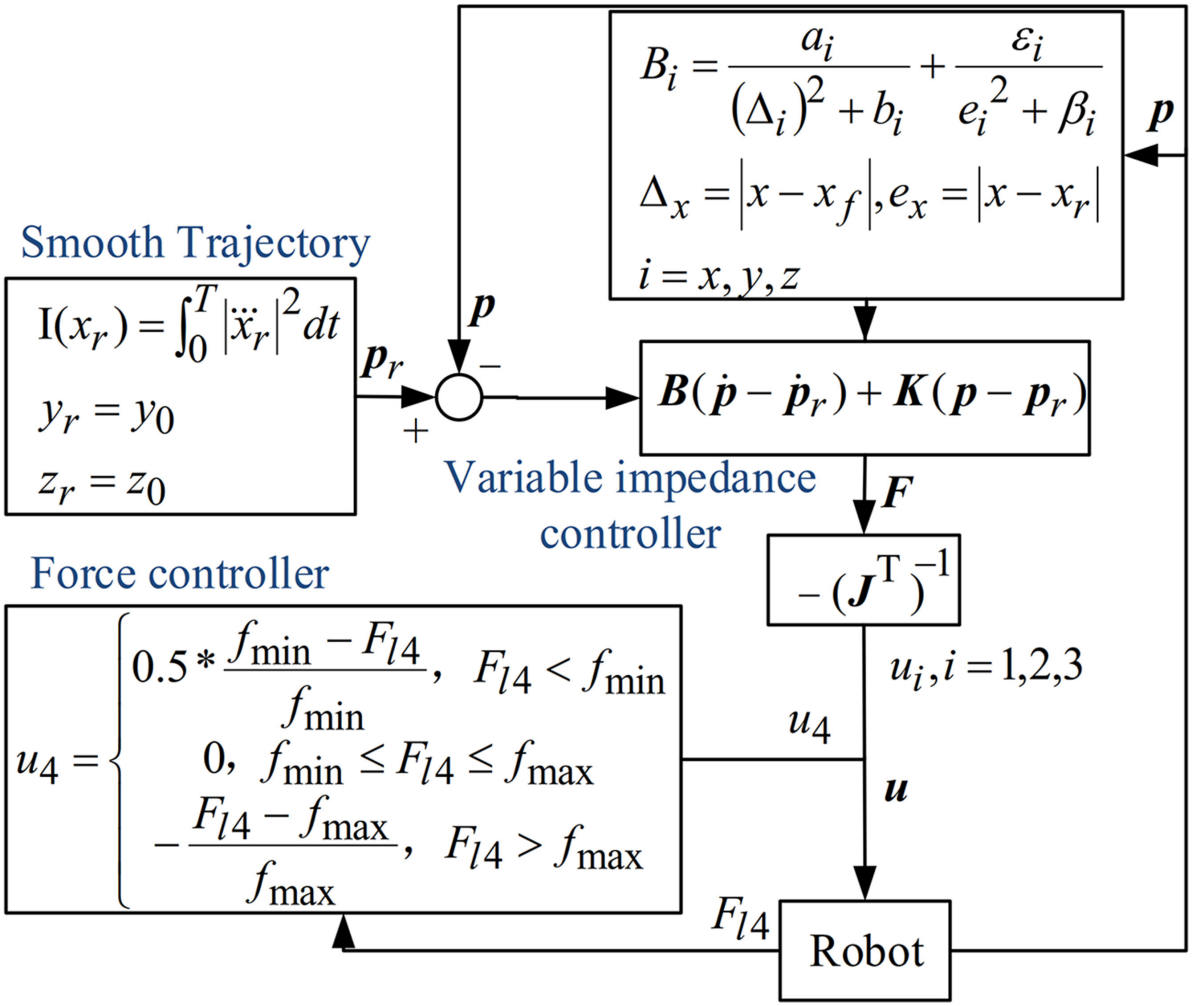
Block diagram of the proposed control algorithm for CDRR.

A hybrid position/force control algorithm was introduced for the CDRR. The block diagram of the control scheme is depicted in Figure 2. The position controller was presented to drive the upper three cables in Figure 1. The downward fourth cable was driven by a force controller. In addition, positive tensile force and negligible elasticity of the cable in CDRR were considered.

In this study, the driven force of the upper three cables was calculated in task space, and then transformed into cable space, so that the end effector could follow the reference trajectory. The impedance dynamics were expressed as follows:


(1) $$\begin{array}{l} M\left(\ddot{p}-{\ddot{p}}_{r}\right)+B\left(\dot{p}-{\dot{p}}_{r}\right)+K\left(p-p_{r}\right)=F \end{array}$$

where ***p*** **=** [*x y z*]^*T*^**∈** ℝ^3^ is the actual position of the end effector in task space; *x, y, z* are the three-dimensional coordinates of the actual end effector at the *i*-th sample. $p_{r}\;={\left[x_{r}\;y_{r}\;z_{r}\right]}^{T}\in \;{\mathbb{R}}^{3}$ is the reference position, and *x*_*r*_, *y*_*r*_, *z*_*r*_ are the reference three-dimensional coordinates of the desired trajectory, respectively. ***M***,***B***,***K*** ∈ ℝ^3×3^ are positive definite diagonal matrices representing the desired virtual inertia, damping, and stiffness; and $F\;={\left[F_{x}\;F_{y}\;F_{z}\right]}^{T}\in \;{\mathbb{R}}^{3}$ represents the control input force.

Since CDRR contributed to a minimized mass on human limbs (Zhong et al., 2022) and the end effector was lightweight, the term of virtual inertia could be ignored. Therefore, the equation 1 was modified as a simplified model retaining stiffness and damping (Chen et al., 2020),


(2) $$\begin{array}{l} B\left(\dot{p}-{\dot{p}}_{r}\right)+K\left(p-p_{r}\right)=F \end{array}$$

In this article, ***B*** **=** *diag*(*B*_*x*_ *B*_*y*_ *B*_*z*_) was changed in real time, while ***K*** **=** *diag*(*K*_*x*_ *K*_*y*_ *K*_*z*_) was a positive constant matrix. Then, the driven force of the upper three cables could be written as follows:


(3) $$\begin{array}{l} u\;=\;-{\left(J^{T}\right)}^{-1}F \end{array}$$

where $u\;=\;{\left[u_{1}\;u_{2}\;u_{3}\right]}^{T}\in \;{\mathbb{R}}^{3}$ is the driven force of the three motors, and $J\;=\;{\left[l_{1}^{T}\;l_{2}^{T}\;l_{3}^{T}\right]}^{T}\in \;{\mathbb{R}}^{3\times \;3}$.

$J_{C}\;\in \;{\mathbb{R}}^{4\times 3}$ represents the Jacobian matrix of CDRR. The details of calculation process are described in equation (Yang et al., 2020).


(4) $$J_{C}\;=\;{\left[l_{1}^{T}\;l_{2}^{T}\;l_{3}^{T}\;l_{4}^{T}\right]}^{T}$$

where **l**_*i*_(*i* = 1, 2, 3, 4) is the length vector along each cable.

For the control of the downward fourth cable, a force controller was given


(5) $$u_{4}=\{\begin{array}{cc} \; & 0.5^{*}\frac{f_{\min}-F_{l4}}{f_{\min}},F_{l4}<f_{\min}\\ \; & 0,\;f_{\min}\leq F_{l4}\leq f_{\max}\\ \; & \;\\ \; & -\frac{F_{l4}-f_{\max}}{f_{\max}},\;F_{l4}>f_{\max} \end{array}$$

where *F*_*l*4_ is the measured tensile force of the fourth cable, *f*_min_, *f*_max_ are threshold values, *f*_min_ was chosen to avoid slackness of the cable, and *f*_max_ was used to reduce the risk of excessive tension of the cable to ensure safety.

### Trajectory Planning

**Figure 3:**
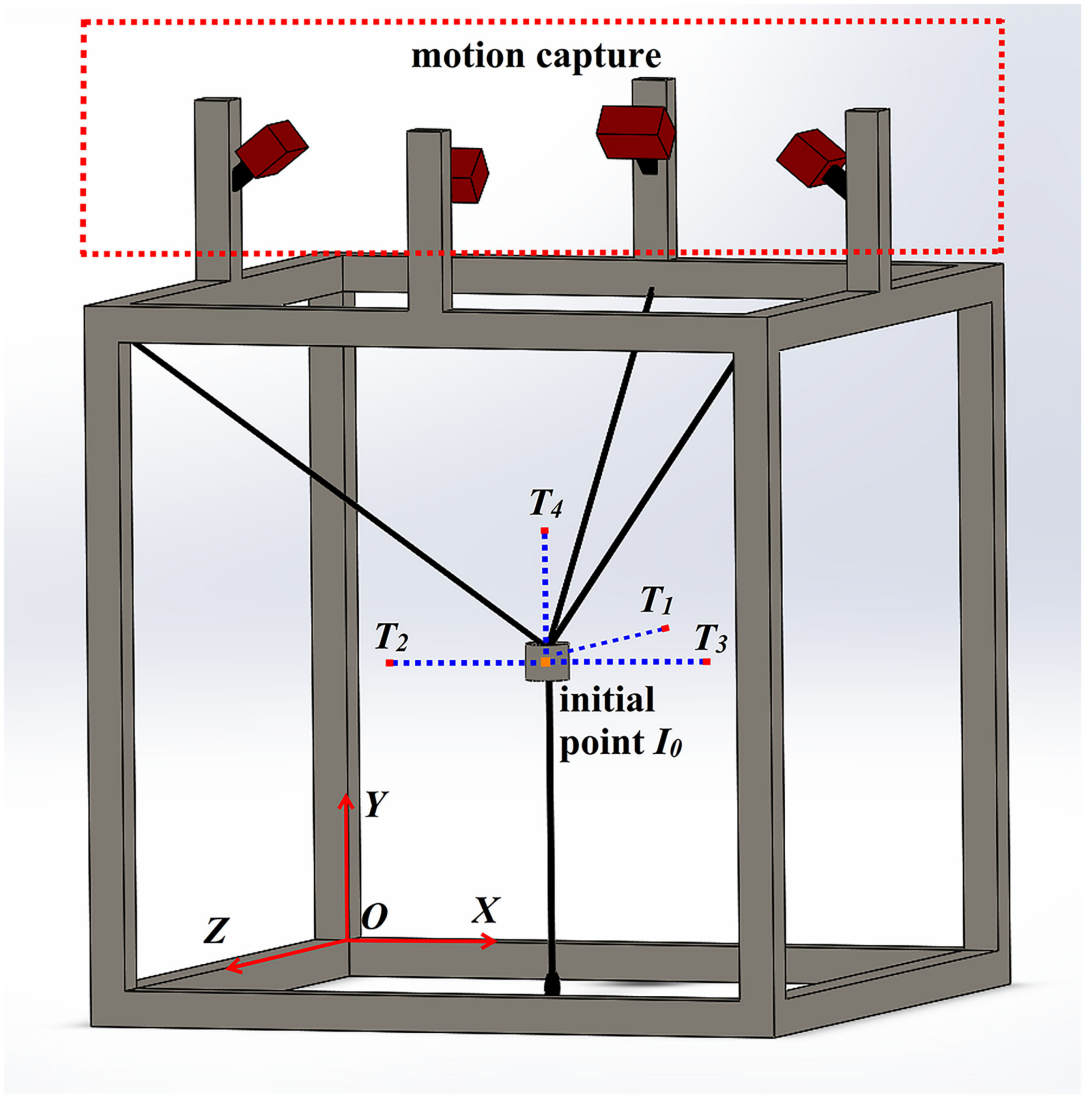
The design of a three-dimensional reaching task, which including four point-to-point tracking subtasks.

The reference trajectory **p**_*r*_ was generated by the MJ model to ensure its smoothness. The proposed controller was applied to a reaching task including four continuous point-to-point tracking subtasks, i.e., forward, rightward, leftward, and upward movements (the target points being *T*_1_, *T*_2_, *T*_3_, *and T*_4_, respectively), as shown in Figure 3. The MJ trajectory was implemented in the main motion axis, whereas the end effector maintained its initial constant value in the other motion axes. If we take a rightward subtask as an example, the reference trajectory of the end effector in the main motion axis, i.e., X axis, was obtained through the MJ model, with initial constant values (*y*_0_, *z*_0_) in the other motion axes, i.e., the Y and Z axes.


(6) $$\begin{array}{l} I\left(x_{r}\right)=\int_{0}^{T}|{\overset{\ldots }{x}}_{r}{|}^{2}dt \end{array}$$

(7) $$\begin{array}{l} {ẋ}_{0}=0,{ẍ}_{0}=0\\ {ẋ}_{f}=0,{ẍ}_{f}=0 \end{array}$$

where *x*_*r*_ is the time series of position in the main motion axis obtained by optimizing equation 6. The minimum jerk trajectory is determined with an initial value *x*_0_, a final value *x*_*f*_, and movement time *T*. The kinematics of the trajectory are constrained by the zero-velocity and zero-acceleration of the initial and final values in equation 7. The following polynomial for the upper-limb reaching movement could be obtained (Flash and Hogan, 1985):


(8) $$\begin{array}{l} x_{r}\left(t\right)=x_{0}+\left(x_{0}-x_{f}\right)\left(-6\tau^{5}+15\tau^{4}-10\tau^{3}\right) \end{array}$$

where $\tau =\frac{t}{T}$. The final position coordinate in *X* axis, *x*_*f*_, was achieved when *t* = *T*.

### Variable Impedance Control

For the variable impedance control in equation 2, the setting of impedance parameters greatly influenced the HRI. When the robot assisted the subject during a certain movement, a non-zero virtual stiffness could be used (Ficuciello et al., 2015). Since the CDRR was a compliant driven robot, variable stiffness could increase the compliance of the robot, which may lead to instability of the system. Unlike the inertia value of a small range, the arm damping value was highly variable (Medina et al., 2019), and mainly influenced by the human perception during the task (Gosselin et al., 2013). In variable impedance control, the variation of impedance with a variable damping and a constant virtual stiffness ensures a better task performance (Li et al., 2018). A damping value modulation law based on the tracking error and the target position was proposed to accommodate the human movement. In this article, the perception distance was processed as from the actual position of the end effector to the target. When the perception distance was shortened or the tracking error was low, the damping value increased (Howard et al., 2013). Prior study has proved that control strategy with a non-linearly changing damping value had better performance results (Stegall et al., 2017). Accordingly, the relationship used to modify the damping value for each of the main motion axis is obtained. Take the *X* axis for example:


(9) $$\begin{array}{l} B_{x}=\frac{a_{x}}{{\left(\Delta_{x}\right)}^{2}+b_{x}}+\frac{\varepsilon_{x}}{{e_{x}}^{2}+\beta_{x}} \end{array}$$

(10) $$\begin{array}{l} \Delta_{x}=|x-x_{f}| \end{array}$$

(11) $$\begin{array}{l} e_{x}=|x-x_{r}| \end{array}$$

where *x*_*f*_ is the target of the subtask, i.e., the final value of the reference trajectory; $\Delta ={\left[\Delta_{x}\;\Delta_{y}\;\Delta_{z}\right]}^{T}$ is the distance between the actual position and the target position; $a={\left[a_{x}\;a_{y}\;a_{z}\right]}^{T},b={\left[b_{x}\;b_{y}\;b_{z}\right]}^{T}$ are constant matrixes related to the perception distance; $\varepsilon ={\left[\varepsilon_{x}\;\varepsilon_{y}\;\varepsilon_{z}\right]}^{T},\beta ={\left[\beta_{x}\;\beta_{y}\;\beta_{z}\right]}^{T}$ are constant matrixes related to the tracking error; $e={\left[e_{x}\;e_{y}\;e_{z}\right]}^{T}$ is the tracking error. The parameters, *a, b*, **ε, β**, are related to the results of impedance control with constant parameters. In the simulation and experiments of the impedance control with appropriate constant parameters, i.e., the value between high and low impedance parameters, the tracking error and the perception distance from the end effector to the target are obtained in real time. Then combing with the range of damping values, the appropriate parameters in equation 9 are calculated. The variable damping values in *Y* and *Z* axes (*B*_*y*_ and *B*_*z*_) are set similarly to the *X* axis.

### Data Analysis

The following parameters, including the evaluation of trajectory tracking accuracy, the trajectory smoothness, and the interaction force, were analyzed.

The tracking accuracy was quantified by the root mean square error (RMSE) and the final error (FE). The RMSE of the tracking error was used to analyze the tracking performance. The RMSE indicated tracking accuracy during the point-to-point reaching task.


(12) $$\begin{array}{l} RMSE=\sqrt{\frac{1}{N}\sum_{i=1}^{N}\left[{\left({x-x}_{r}\right)}^{2}+{\left({y-y}_{r}\right)}^{2}+{\left({z-z}_{r}\right)}^{2}\right]} \end{array}$$

where *N* is the number of samples. The FE was defined as the mean tracking error maintained at the endpoint for 2 s after reaching the target.

The shakiness and normalized jerk score (NJS) were used to analyze the trajectory smoothness. The shakiness was regarded as the number of zero-crossings for the acceleration profile in the main motion axis. A lower shakiness value demonstrated a smoother tracking trajectory. NJS was calculated by a normalized jerk while removing the effect of the time and amplitude of the movement.


(13) $$\begin{array}{l} NJS=\sqrt{\frac{1}{2}\cdot \frac{T^{5}}{\alpha^{2}}\cdot \int {\left(jerk\right)}^{2}dt} \end{array}$$

where *T* is the movement time, α is the movement amplitude, and *jerk* is the third derivative of the actual position. A lower NJS value indicated less jerkiness.

The mean interaction force was calculated for each task. Since the simulation was implemented in the task space, the mean output driven force was calculated in three-dimensional coordinates. In the experiment, the output force in the *X, Y*, and *Z* axes was translated into the force of each cable through the inverse Jacobian matrix. After this, the mean resultant force during the movements was obtained.

The mean value of the five evaluation indicators of different control algorithms were calculated across each trial. All the indicators were described using the mean ± standard error (SE). The paired *t*-tests with a significance level of 0.05 were used to analyze the effect of the control algorithms.

## Simulation and Experiment

### Simulation Setup

Simulation was performed with the software MATLAB to verify the effectiveness of the proposed variable impedance control (vIC) during a rightward subtask. The following control algorithms were also conducted for comparison: a) impedance control with constant parameters (IC) where both constant high and low damping values were considered (IC-H, IC-L), b) the variable impedance control in which the damping value was only changed based on the tracking error (vIC-e). As a result, the relationship used to vary the damping value in the task space was obtained. Take the *X* axis for example:


(14) $$\begin{array}{l} B_{x}=\frac{\epsilon_{x}}{{e_{x}}^{2}+\beta_{x}} \end{array}$$

where $\epsilon ={\left[\epsilon_{x}\;\epsilon_{y}\;\epsilon_{z}\right]}^{T}$ is a constant matrix related to the tracking error. This parameter **ε, β** in equation 14 is calculated similarly to equation 9. The variable damping values and parameters in the *Y* and *Z* axes are calculated in similar way to that of the *X* axis. The desired trajectory of the vIC-e was the same as that of the vIC.

Only a rightward subtask was considered as a representative in the simulation. In the simulation, the damping value of the vIC varied between the minimum (0.7 Ns/m) and the maximum (1.1 Ns/m) values according to Δ*x* and *e* in equation 9. The variable damping was compared with the case of IC-H and IC-L with maximum [*B* = *diag*(1.1 1.1 1.1)] and minimum [*B* = *diag*(0.7 0.7 0.7)] values, respectively. A MJ trajectory from an initial position *I*_0_ = (0 0 0 ) to a final position *T*_*f*_ = (0.2 0 0) in a movement time (*T* = 6 s) was set. To take the effect of the human into account, a sinusoidal disturbance with an amplitude of 0.001 m and a frequency of 5 Hz was introduced during the movement. The parameters related to the damping and stiffness in the simulation are given in Table 1.

**Table 1:** Parameters of the simulation.

	**Parameter**	**Value**
vIC	***a***	[5.39 × 10^−2^ 8.42 × 10^−10^ 8.42 × 10^−10^]^*T*^
	***b***	[7.00 × 10^−2^ 1.09 × 10^−9^ 1.09 × 10^−9^]^*T*^
	**ε**	[5.65 × 10^−8^ 3.61 × 10^−10^ 3.61 × 10^−10^]^*T*^
vIC-e	**ϵ**	[1.88 × 10^−7^ 3.61 × 10^−10^ 3.61 × 10^−10^]^*T*^
	**β**	[1.71 × 10^−7^ 1.09 × 10^−9^ 1.09 × 10^−9^]^*T*^
	***K***	*diag*(40 40 40)

### Experimental Setup

Five healthy subjects (three females, two males; age: 23.6 ± 0.5 years; height: 168.8 ± 5.9 cm; weight: 55.6 ± 5.1 kg) were recruited to participate in the experiment, which was conducted on the CDRR. All the subjects signed the informed consent forms, and the experiments were approved by Sun Yat-Sen University. 5 min were given to each subject before the experiment to be familiar with the tasks. 1 min was given for a rest every reaching task in order to reduce arm fatigue.

During the experiment, the subject was asked to sit in front of the end effector. The CDRR moved the user's hand to fulfill a designed task with the assistance of the control algorithms. In Figure 3, the task consisted of four continuous point-to-point tracking subtasks: forward, leftward, rightward, and upward movements. The coordinate of the initial point was *I*_0_ = (0.06 0.9 − 0.4), and the target points were *T*_1_ = (0.06 0.9 − 0.6), *T*_2_ = (0.26 0.9 − 0.4), *T*_3_ = (−0.14 0.9 − 0.4), and *T*_4_ = (0.06 1.1 − 0.4). The length of each target trajectory was 0.2 m, and the movement time for each subtask was 6 s (*T* = 6 s). 3 s were taken to stay at the endpoint after reaching the target. The control algorithm was performed five times for each subtask. During the experiment, a screen comprising a virtual environment was placed in front of the subject. And the three-dimensional real-time desired path and the actual position of the end effector were shown in the virtual environment using virtual cursors. The experiments with following control algorithms were implemented: (a) IC-H, (b) IC-L, (c) vIC, and (d) vIC-e. In the experiments, the high constant damping value of IC-H was *B* = *diag*(7 7 7), whereas the low constant damping value of IC-L was *B* = *diag*(1 1 1). Other parameters are shown in Table 2.

**Table 2:** Parameters of the experiments.

	**Parameter**	**Value**	
		**Rightward**	**Leftward**
vIC	***a***	[3.27 × 10^−2^ 9.88 × 10^−5^ 1.60 × 10^−4^]^*T*^	[3.27 × 10^−2^ 9.10 × 10^−5^ 2.16 × 10^−4^]^*T*^
	***b***	[6.70 × 10^−3^ 2.02 × 10^−5^ 3.27 × 10^−5^]^*T*^	[6.70 × 10^−3^ 2.60 × 10^−6^ 6.16 × 10^−5^]^*T*^
	**ε**	[4.23 × 10^−5^ 4.21 × 10^−5^ 6.84 × 10^−5^]^*T*^	[3.91 × 10^−5^ 3.85 × 10^−5^ 9.23 × 10^−5^]^*T*^
	**β**	[2.01 × 10^−5^ 1.94 × 10^−5^ 3.21 × 10^−5^]^*T*^	[2.62 × 10^−6^ 2.42 × 10^−5^ 6.11 × 10^−5^]^*T*^
vIC-e	**ϵ**	[1.41 × 10^−4^ 1.16 × 10^−4^ 2.28 × 10^−4^]^*T*^	[1.49 × 10^−4^ 1.20 × 10^−4^ 2.62 × 10^−4^]^*T*^
	**β**	[2.01 × 10^−5^ 1.59 × 10^−5^ 3.21 × 10^−5^]^*T*^	[2.10 × 10^−5^ 1.57 × 10^−5^ 3.71 × 10^−5^]^*T*^
	***K***	*diag*(80 30 30)	*diag*(80 30 30)
		**Forward**	**Upward**
vIC	***a***	[7.35 × 10^−6^ 1.60 × 10^−4^ 3.27 × 10^−2^]^*T*^	[1.06 × 10^−5^ 3.27 × 10^−2^ 8.36 × 10^−6^]^*T*^
	***b***	[1.50 × 10^−6^ 3.27 × 10^−5^ 6.70 × 10^−3^]^*T*^	[2.16 × 10^−6^ 6.70 × 10^−3^ 1.71 × 10^−6^]^*T*^
	**ε**	[2.87 × 10^−6^ 6.30 × 10^−5^ 3.47 × 10^−5^]^*T*^	[4.31 × 10^−6^ 3.68 × 10^−5^ 3.55 × 10^−6^]^*T*^
	**β**	[5.55 × 10^−7^ 1.40 × 10^−5^ 1.55 × 10^−5^]^*T*^	[1.41 × 10^−6^ 1.50 × 10^−6^ 1.60 × 10^−6^]^*T*^
vIC-e	**ϵ**	[1.33 × 10^−5^ 2.10 × 10^−4^ 1.31 × 10^−4^]^*T*^	[1.44 × 10^−5^ 1.23 × 10^−4^ 1.18 × 10^−5^]^*T*^
	**β**	[1.10 × 10^−6^ 1.40 × 10^−5^ 9.67 × 10^−6^]^*T*^	[1.41 × 10^−6^ 1.50 × 10^−6^ 1.60 × 10^−6^]^*T*^
	***K***	*diag*(30 30 80)	*diag*(30 80 30)

## Results

### Simulation Results

**Figure 4:**
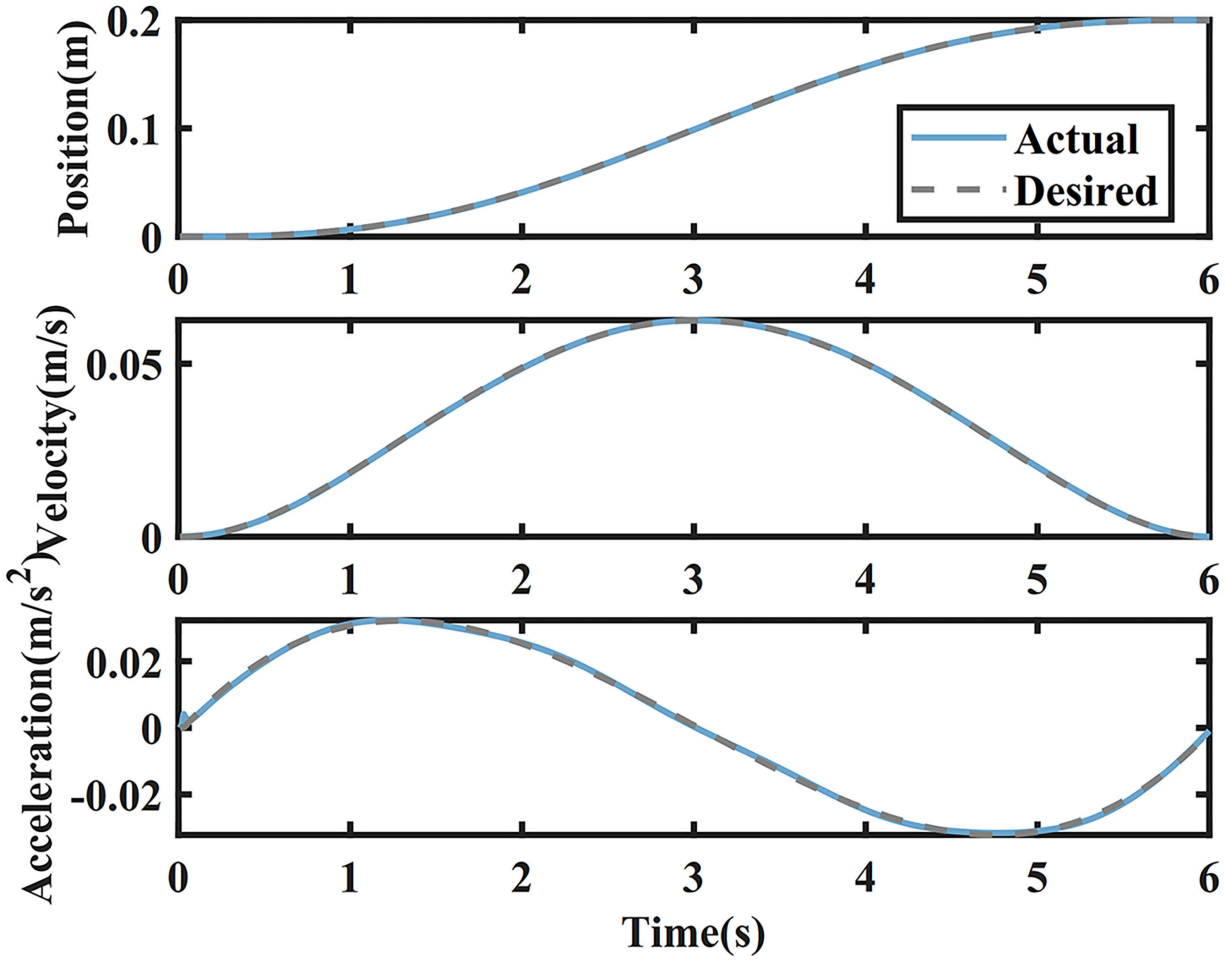
The desired and actual values of the trajectory, velocity, and acceleration of the impedance control with high constant damping (IC-H) in the main motion axis.

**Figure 5:**
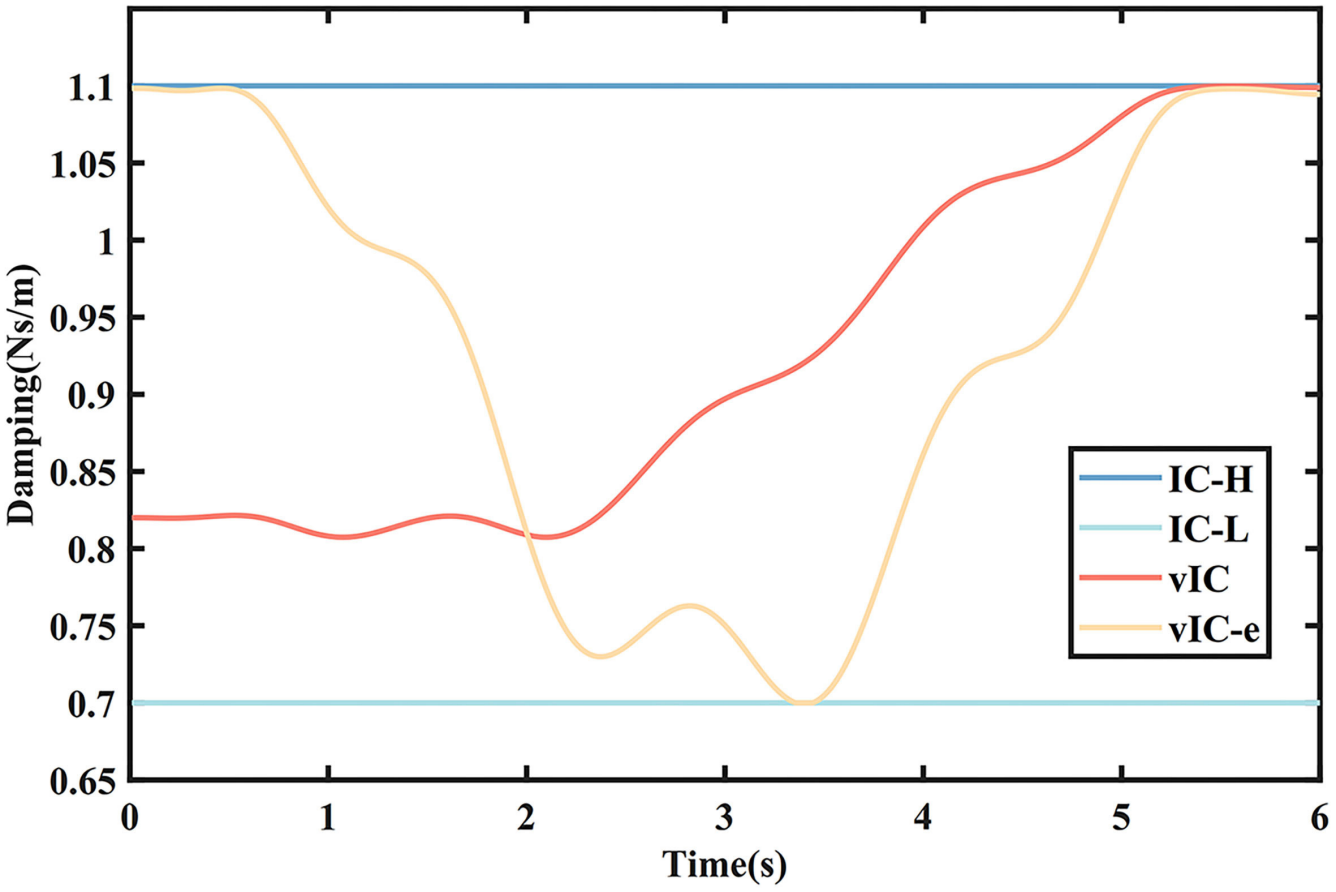
The damping values of different control algorithms in the main motion axis.

The simulation results of IC, vIC, and vIC-e are shown in Figures 4, 5. The tracking position, velocity, and acceleration of IC-H are depicted in Figure 4. The tracking performances of vIC and vIC-e were similar with those of IC, and are not shown. It was found that the actual trajectory coincided with the desired one; the bell-shaped velocity profile conformed to the characteristics of the MJ trajectory; and the actual acceleration changed continuously and gently with little oscillation in the initial phase.

The damping values of different control algorithms are depicted in Figure 5. The damping value of vIC was higher in the initial phase, decreased slightly, and increased gradually when it was close to the target. The damping value of vIC-e changed more acutely, with a minimum in the middle of the movement, and a relatively high level in both the initial and final phase.

The RMSE, FE, shakiness, NJS, as well as mean output force of IC, vIC, and vIC-e are listed in Table 3. For the tracking accuracy, lower RMSE and FE were obtained when the damping value was higher. That is, lower RMSE and FE in IC-H were found than those of IC-L. The RMSE and FE values of IC were intermediate compared to those of IC-H and IC-L. The results of RMSE were similar between vIC and vIC-e; but the value of FE was larger for vIC-e than that of vIC. In terms of trajectory smoothness, the shakiness was the same for different control algorithms. A lower NJS was found in IC-H than IC-L. And lower NJS was obtained for vIC compared with those of IC and vIC-e. The mean interaction force of vIC was the same as that of IC-L, but larger than that of IC-H. And vIC-e had a slightly smaller mean force than vIC.

**Table 3:** Indicators of IC, vIC, and vIC-e in simulation.

		**Tracking accuracy**		**Trajectory smoothness**		**Interaction force**
**B**		**RMSE**	**FE**	**Shakiness**	**NJS**	**Mean force**
**(Ns/m)**		**(×10^−2^m)**	**(×10^−3^m)**		**(×10^2^)**	**(×10^−3^*N*)**
IC	1.1	3.3096	4.0139	1	1.1564	6.3891
	0.7	3.5394	4.1440	1	1.1645	6.3975
vIC	—	3.3450	4.0143	1	1.1558	6.3975
vIC-e	—	3.3103	4.2245	1	1.1572	6.3922

### Experimental Results

**Figure 6:**
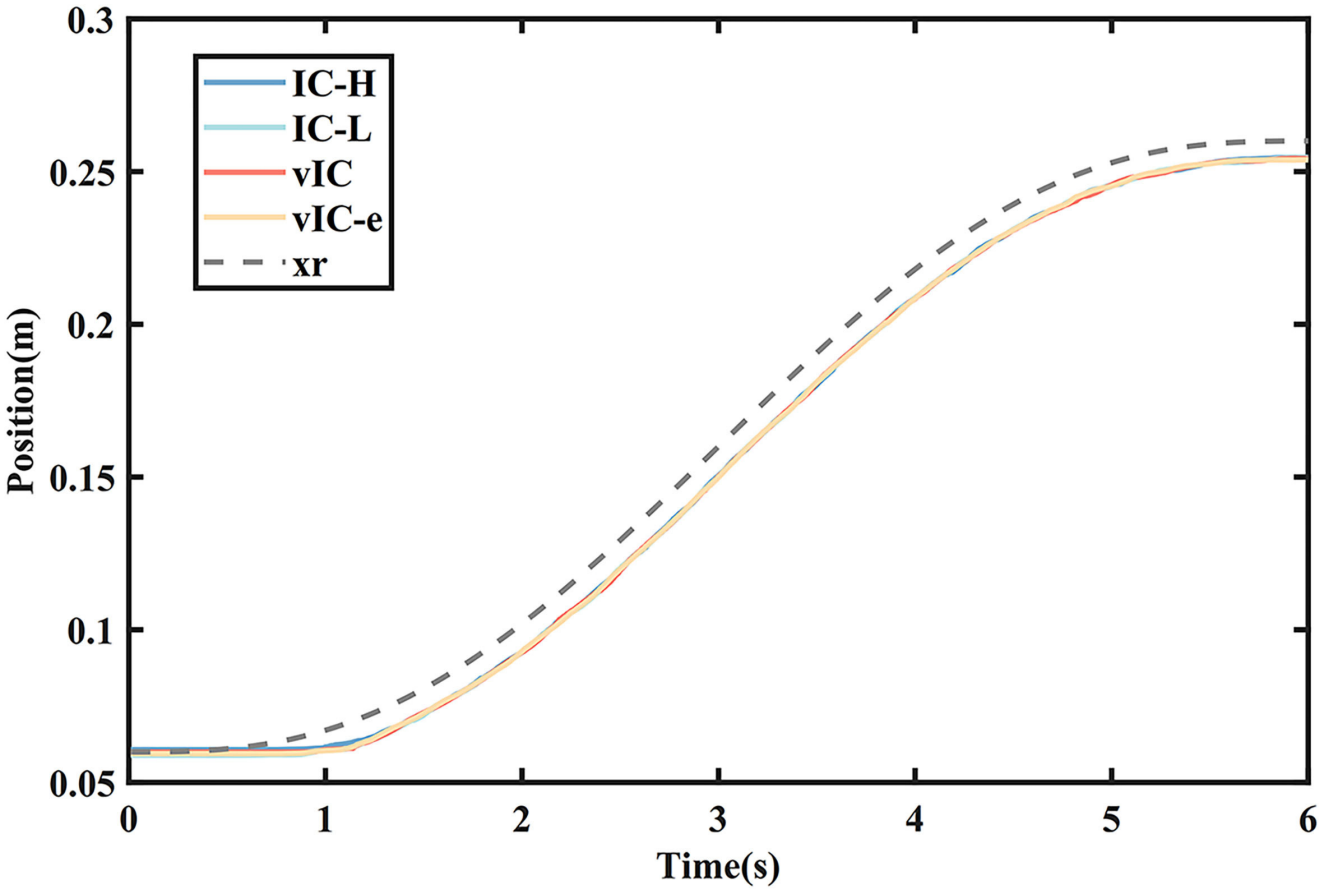
The actual (solid lines) and desired (gray dashed line) trajectory profile of four control algorithms during a rightward subtask.

**Figure 7:**
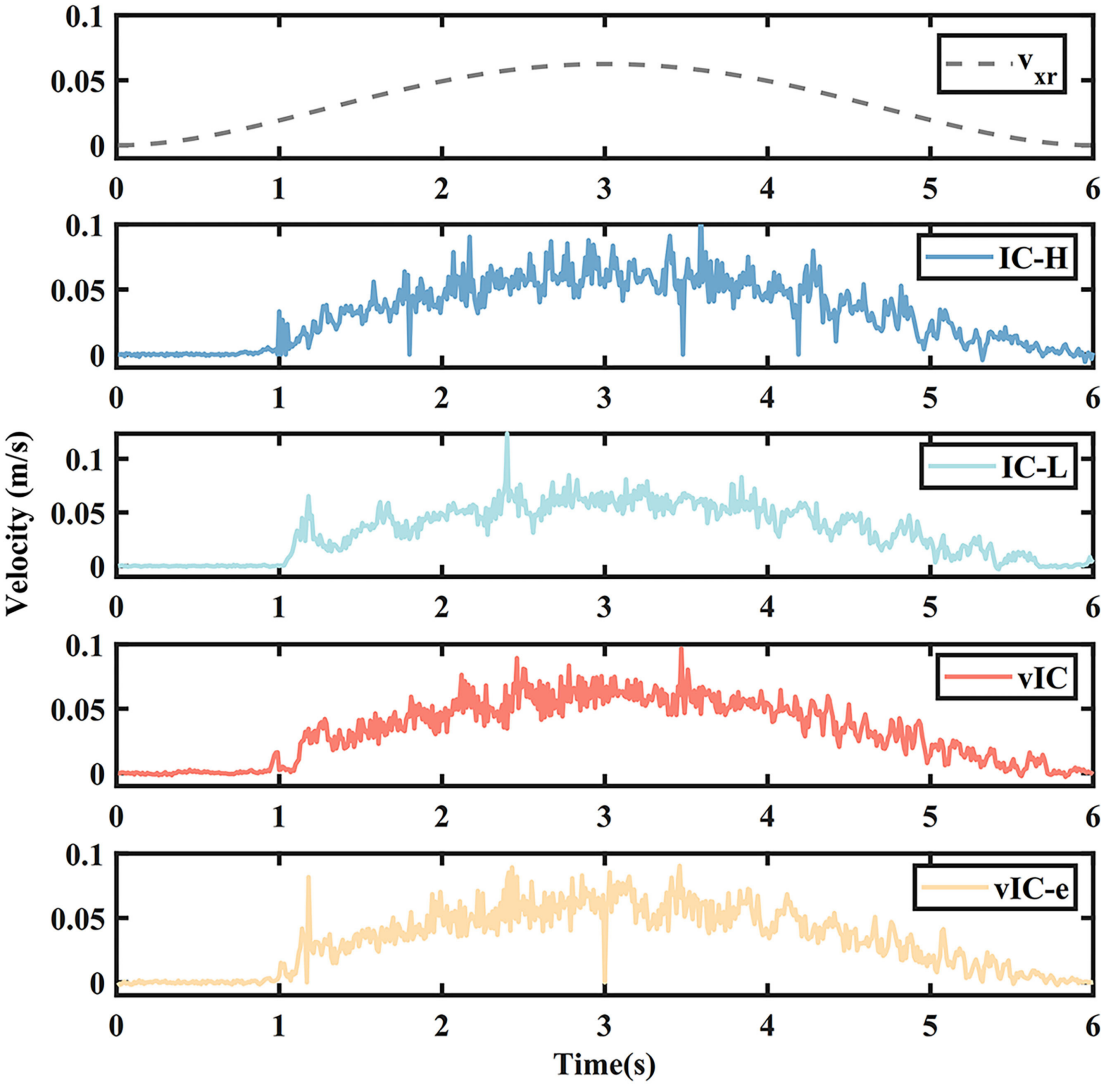
The actual (solid lines) and desired (gray dashed line) velocity profile of four control algorithms during a rightward subtask.

**Figure 8:**
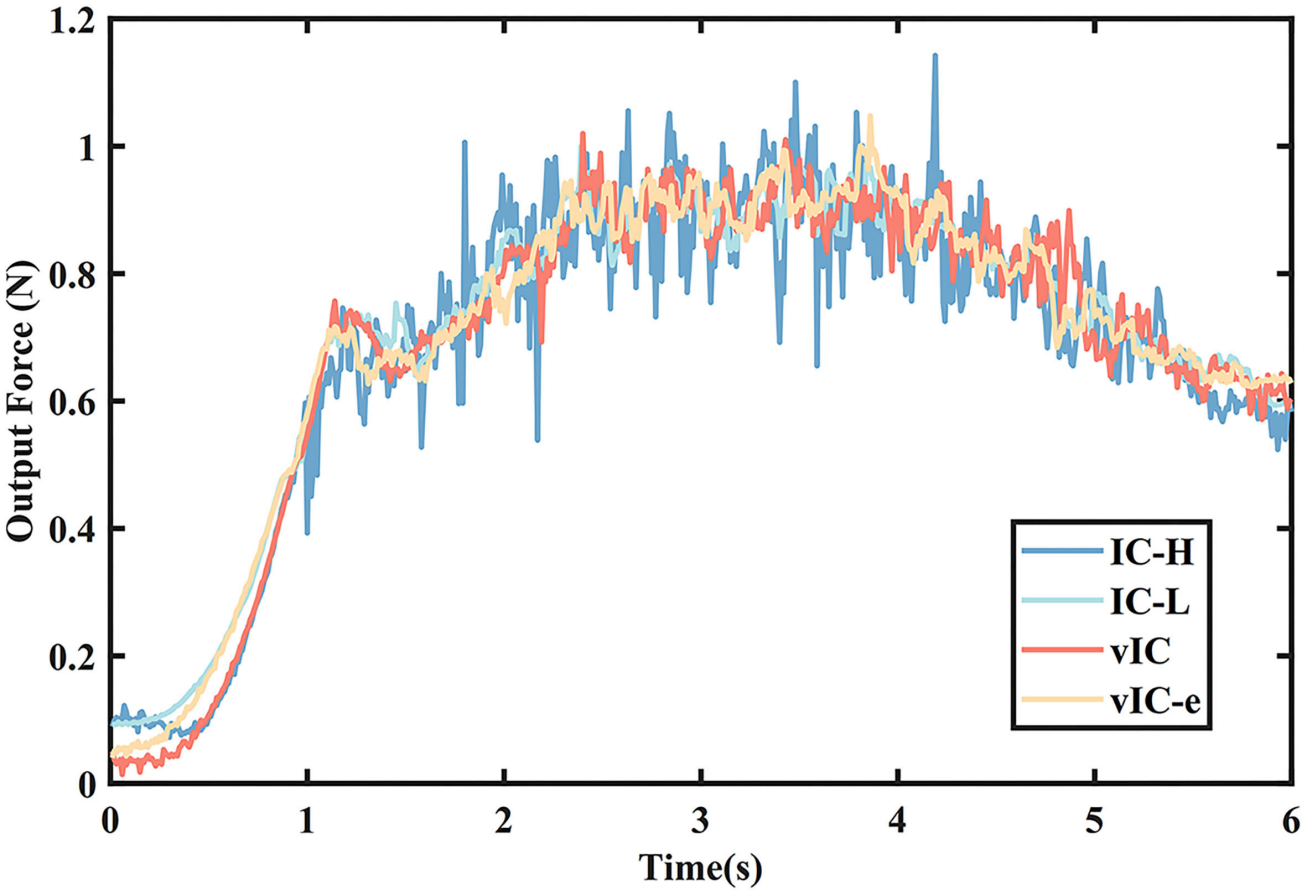
The mean output force of four control algorithms during a rightward subtask.

**Figure 9:**
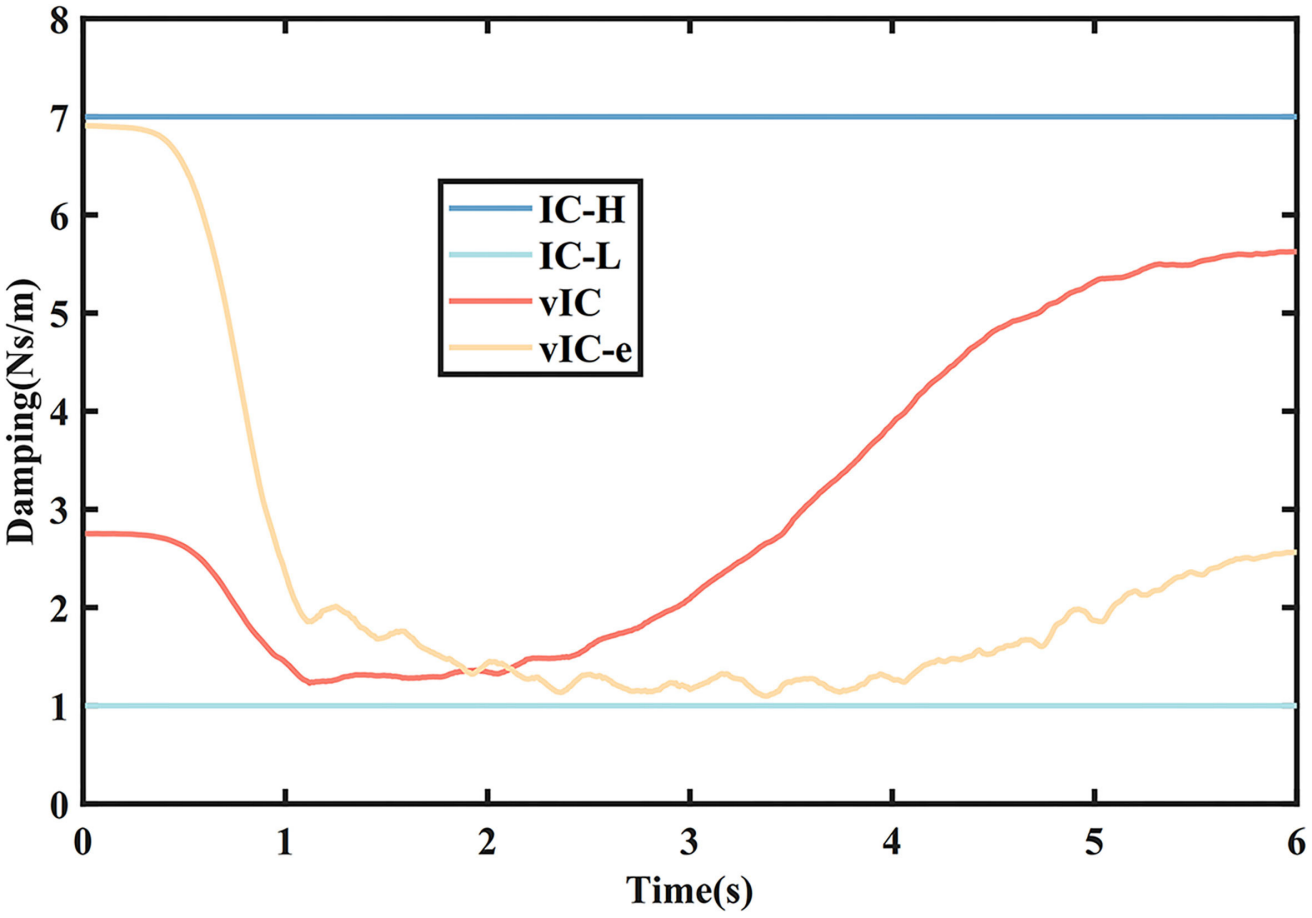
The damping values of four control algorithms in the main motion axis during a rightward subtask. The maximum of damping values is 7 Ns/m (IC-H), while the minimum is 1 Ns/m (IC-L).

Taking the representative rightward subtask as an example, that is, from *I*_0_ to *T*_2_, the measured data are given in Figures 6–**9**. The actual and desired trajectory of IC-H, IC-L, vIC, and vIC-e in the main motion axis are shown in Figure 6. It was found that the robot with the four control algorithms could follow the desired trajectory accurately.

The actual and desired velocity profile of the four control algorithms in the main motion axis are depicted in Figure 7. The overall actual velocity profiles of IC-H, IC-L, vIC, and vIC-e were bell-shaped, which conformed to the characteristics of the MJ trajectory. The actual velocity profile of IC-H had a severe fluctuation and several “spikes” during the movement; and the velocity profile of IC-L fluctuated fiercely in the initial and second half phase. The velocity profile of vIC showed a small fluctuation at all stages. Compared to vIC, the velocity profile of vIC-e fluctuated more fiercely, and several large “spikes” were observed. Overall, the velocity profile of vIC changed more gradually within a narrow range.

The mean output forces of the four control algorithms are presented in Figure 8. It was shown that the mean force of vIC was lower than that of IC at the beginning of the motion, and then it increased with an intermediate value between those of IC-H and IC-L, and ultimately remained at a relative low value when the end-effector was close to the target. The mean force of vIC-e was higher than that of vIC in the first half phase, and similar to that of vIC in the second half phase.

Figure 9 shows the actual damping values of the four control algorithms during a rightward subtask. The damping value of vIC changed gradually at all stages. It was medium at the beginning of the motion to remain stable and reduce the impact; then it decreased to ensure a quick start; and eventually it increased to reach the target smoothly and accurately. By comparison, the damping value of vIC-e changed with relatively large fluctuation, for it only varied with the tracking error. The damping value of vIC-e was relatively high as a result of small tracking error at the beginning of the motion; and was lower than that of vIC due to the existence of the tracking error when reaching the target.

**Figure 10:**
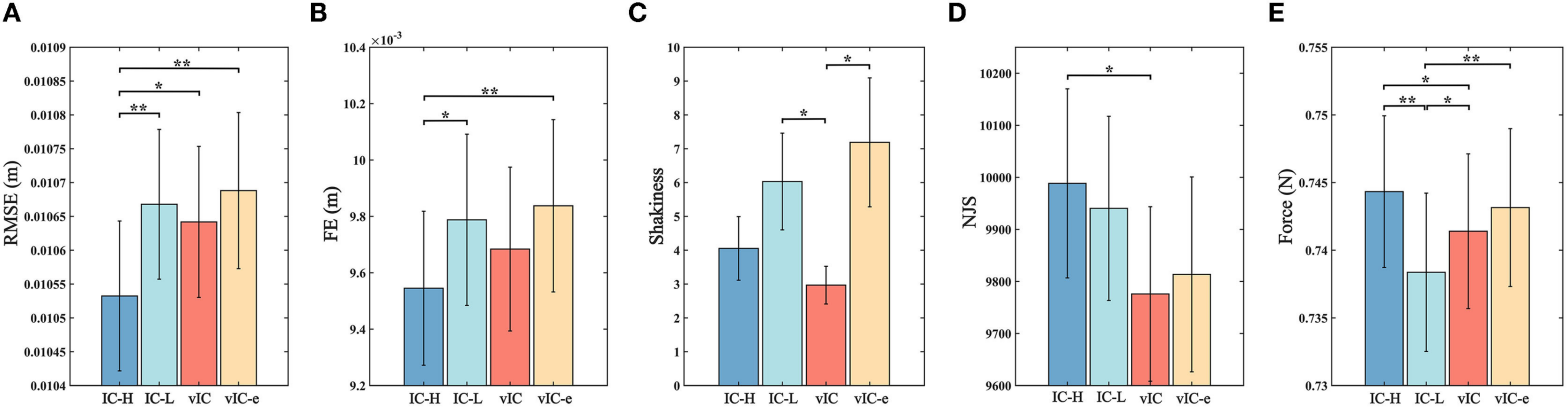
The analysis parameters of four control algorithms for a reaching task. Average parameter for the four point-to-point tracking subtasks (mean ± SE). **(A)** Statistical results of the root mean square error (RMSE) for all subjects. **(B)** Statistical results of four control algorithms expressed the final error (FE). **(C)** Analysis of the shakiness. **(D)** Analysis of the normalized jerk score (NJS). **(E)** Analysis of the mean output force in the experiments. *Indicates significant difference between two control algorithms at 0.05 level; and **Indicates significant difference between two control algorithms at 0.01 level.

The mean value of the five parameters of different control algorithms for all the four subtasks during the experiment are summarized in Figure 10. The tracking accuracy of different control algorithms was compared in Figures 10A,B. The RMSE of IC-H was the smallest among different control algorithms. The RMSE value of vIC was intermediate between those of IC-H and IC-L, and lower than that of vIC-e. Significant differences were found in RMSE between IC-H and other control algorithms. The lowest FE was achieved for IC-H, which indicated that the target was hit accurately. Significant differences were found in FE between IC-H and IC-L, vIC-e. The FE of vIC was intermediate between those of IC-H and IC-L, and lower than that of vIC-e, although it was not statistically significant. In terms of trajectory smoothness (Figures 10C,D), the shakiness of vIC was the smallest. And significant differences were found between vIC and IC-L, vIC-e. Similarly, the NJS of vIC was the smallest. And significant difference was found between vIC and IC-H. As for the mean output force, the IC-H had the highest value; IC-L was the lowest among the four control algorithms; and the mean output force of vIC was lower than that of vIC-e. Significant differences were found between IC-H and IC-L, vIC, between IC-L and vIC, vIC-e. In summary, it was observed that the vIC improved the trajectory smoothness and achieved a good compromise between IC-H and IC-L in tracking accuracy and interaction force, and outperformed the vIC-e.

## Discussion

The proposed variable impedance control algorithm with CDRR was designed for rehabilitation training to achieve accurate and smooth tracking performance, as well as compliant HRI. The control algorithm in this article included variable impedance and smooth trajectory, which was in accord with the characteristics of the human upper-limb during the reaching movement (Ajoudani et al., 2012). The variable impedance was related to the target position and the tracking error; whereas the smooth reference trajectory was guaranteed by the MJ model.

In this article, a hybrid position/force control algorithm was implemented on the CDRR. The position controller of the upper three cables was used to track the reference trajectory and fulfill the reaching task. And the force controller of the downward forth cable guaranteed safe HRI and the stability of the system. The hybrid position/force control algorithm redistributed the tensile force of each cable and made the tension in an appropriate range. It was a relatively complex problem due to the under-constraints if only the position controller was used. Besides, the coupling between each motor might cause excessive cable tensile force, which might cause a safety hazard to the subjects. Yasin and Simaan used a hybrid force/position control algorithm for a medical robot to achieve safe HRI (Yasin and Simaan, 2021). Likewise, the hybrid position/force control algorithm in this article could ensure safe HRI as well as good tracking performance.

The results of both the simulations and experiments showed that the tracking accuracy and robustness of the system was ensured when the impedance gain value was set to a constant large value (Lewis et al., 2004). Due to the high damping value in the initial and final phase, the tracking accuracy indicators of vIC was between those of IC-H and IC-L, which was in accord with the results of Dong and Ren (Dong and Ren, 2019) with sinusoidal impedance parameters. On the contrary, the damping value of vIC-e was maintained at a relatively low level in the final phase, which went against the possibility of placidly reaching the target and its steady maintenance. It might be the reason for the better tracking accuracy of vIC compared to the vIC-e. In addition, the actual trajectory smoothness of vIC was better than those of both IC and vIC-e as the vIC was able to adjust its damping value in a more appropriate way according to the needs. In comparison, the damping value of vIC-e was only dependent on the tracking error that fluctuated greatly, which resulted in poor tracking smoothness. In terms of the neurophysiological view, the term of the target position in equation 9 was regarded as motion feedforward, and the term of the tracking error was viewed as motion feedback. Therefore, vIC could dynamically adjust the damping more appropriately based on both the target position and the tracking error. Though a larger impedance value could improve the robot's performance in tracking accuracy, but the interaction force was relatively large. By contrast, a lower impedance value had lower interaction force but poor tracking accuracy. The results of IC-H and IC-L in this article agreed well with the finding of Mersha et al. (2014). The force of vIC was intermediate between that of IC-H and IC-L, which was consistent with the findings of Ficuciello et al. (2015) whose damping parameter was modified based on the velocity of the movement. The mean force of vIC-e was higher compared with vIC, which might lead to unfriendly HRI.

The vIC performed better than IC in terms of trajectory smoothness, and displayed medium tracking accuracy and interaction force with respect to constant low and high damping values. And with respect to vIC-e, better performance of vIC was obtained in all respects. The results in this article also might demonstrate the assumption that a control strategy with human-like characteristics could ensure accurate and smooth tracking performance, as well as safe and natural HRI (Roesler et al., 2021).

Repetitive rehabilitation training is required for patients who have motor dysfunction (Hussain et al., 2018). In order to prevent severe movement disorder and muscular dystrophy (Sommerfeld et al., 2004), early passive training is essential to improve motor control ability (Ren et al., 2017). Smooth and compliant exercise training is more effective for the rehabilitation of motor function (Zhang et al., 2018). The smooth trajectory and variable impedance parameter of vIC made the robot humanlike, which was more acceptable for the subject. Hence, it is predicted that the robot with the proposed control algorithm might improve the rehabilitation effectiveness in the following clinical applications.

There are a couple of limitations in this study that should be addressed. The subjects in this article are physically healthy. The proposed method is mainly aimed at point-to-point reaching tasks. Since the MJ trajectory requires the awareness of the movement time in advance (Hogan, 1984), the reference trajectory is incompatible for uncertain motion. The protocol of the cable-driven rehabilitation robot is being refined to satisfy the needs of patients with upper-limb impairment further. In future research, patients will be recruited to validate the clinical effectiveness of the proposed controller. And different tasks and difficulty of the movement and other human motion models might be considered. And it is important to consider trajectory shaping on kinematics and biological parameters based on human-like characteristics in order to improve HRI.

## Conclusion

In this article, the variable impedance control algorithm with human-like characteristics was proposed for a rehabilitation robot. The simulation and experimental results of a point-to-point reaching task showed that the proposed method could fulfill the designed task with a more compliant and natural form of human-robot interaction. The proposed method thereby might exhibit promising potential in terms of clinical application.

## Data Availability Statement

The raw data supporting the conclusions of this article will be made available by the authors, without undue reservation.

## Ethics Statement

The studies involving human participants were reviewed and approved by the Ethics Committee of the Sun Yat-sen University. The patients/participants provided their written informed consent to participate in this study.

## Author Contributions

QY and RS conceived and designed the study and reviewed and edited the manuscript. RT performed the experiments and wrote the article. All the authors had read and approved the final version of the manuscript.

## Funding

This study was supported by the Natural Science Foundation of China (Grant No. 61803395), the Guangdong Basic and Applied Basic Research Foundation (Grant No. 2021A1515012339), the Guangdong Natural Science Foundation (Grant No. 2018A030310280), the Fundamental Research Funds for the Central Universities (Grant No. 18zxxt27), the National Key Research and Development Program of China (Grant No. 2018YFC2001600), the Shenzhen Science and Technology Plan Project (Grant No. GJHZ20200731095211034), and the Guangdong Science and Technology Plan Project (Grant No. 2020B1212060077).

## Conflict of Interest

The authors declare that the research was conducted in the absence of any commercial or financial relationships that could be construed as a potential conflict of interest.

## Publisher's Note

All claims expressed in this article are solely those of the authors and do not necessarily represent those of their affiliated organizations, or those of the publisher, the editors and the reviewers. Any product that may be evaluated in this article, or claim that may be made by its manufacturer, is not guaranteed or endorsed by the publisher.
